# DNApod: DNA polymorphism annotation database from next-generation sequence read archives

**DOI:** 10.1371/journal.pone.0172269

**Published:** 2017-02-24

**Authors:** Takako Mochizuki, Yasuhiro Tanizawa, Takatomo Fujisawa, Tazro Ohta, Naruo Nikoh, Tokurou Shimizu, Atsushi Toyoda, Asao Fujiyama, Nori Kurata, Hideki Nagasaki, Eli Kaminuma, Yasukazu Nakamura

**Affiliations:** 1 Genome Informatics Laboratory, National Institute of Genetics, Mishima, Shizuoka, Japan; 2 Database Center for Life Science, Joint Support-Center for Data Science Research, Research Organization of Information and Systems, Mishima, Shizuoka, Japan; 3 Department of Liberal Arts, The Open University of Japan, Chiba, Chiba, Japan; 4 Division of Citrus Research, Institute of Fruit Tree and Tea Science, NARO, Shimizu, Shizuoka, Japan; 5 Comparative Genomics Laboratory, National Institute of Genetics, Mishima, Shizuoka, Japan; 6 Advanced Genomics Center, National Institute of Genetics, Mishima, Shizuoka, Japan; 7 Plant Genetics Laboratory, National Institute of Genetics, Mishima, Shizuoka, Japan; 8 Genome Informatics Group, Department of Technology Development, Kazusa DNA Research Institute, Kisarazu, Chiba, Japan; Montana State University Bozeman, UNITED STATES

## Abstract

With the rapid advances in next-generation sequencing (NGS), datasets for DNA polymorphisms among various species and strains have been produced, stored, and distributed. However, reliability varies among these datasets because the experimental and analytical conditions used differ among assays. Furthermore, such datasets have been frequently distributed from the websites of individual sequencing projects. It is desirable to integrate DNA polymorphism data into one database featuring uniform quality control that is distributed from a single platform at a single place. DNA polymorphism annotation database (DNApod; http://tga.nig.ac.jp/dnapod/) is an integrated database that stores genome-wide DNA polymorphism datasets acquired under uniform analytical conditions, and this includes uniformity in the quality of the raw data, the reference genome version, and evaluation algorithms. DNApod genotypic data are re-analyzed whole-genome shotgun datasets extracted from sequence read archives, and DNApod distributes genome-wide DNA polymorphism datasets and known-gene annotations for each DNA polymorphism. This new database was developed for storing genome-wide DNA polymorphism datasets of plants, with crops being the first priority. Here, we describe our analyzed data for 679, 404, and 66 strains of rice, maize, and sorghum, respectively. The analytical methods are available as a DNApod workflow in an NGS annotation system of the DNA Data Bank of Japan and a virtual machine image. Furthermore, DNApod provides tables of links of identifiers between DNApod genotypic data and public phenotypic data. To advance the sharing of organism knowledge, DNApod offers basic and ubiquitous functions for multiple alignment and phylogenetic tree construction by using orthologous gene information.

## Introduction

Genome-wide DNA polymorphism datasets are powerful tools that help to resolve biological questions. With the development of microarray and next-generation sequencing (NGS) technologies, genome-wide DNA polymorphisms have been studied intensively for the past 10 years. DNA polymorphisms can affect the phenotype of an organism and are useful as DNA markers, and a combination of genome-wide DNA marker sets and phenotypic data gathered for populations can be used to reveal loci underlying phenotypes through genome-wide association studies (GWAS) [1–3]. Moreover, the combination of genome-wide DNA marker and phenotype datasets is used in breeding programs, and modern programs have adopted the marker-assisted selection (MAS) approach. However, MAS frequently fails to identify quantitative trait loci that produce small effects. For overcoming this drawback, genomic selection, which predicts phenotypic information based on high-density DNA markers, has received attention as a useful technology for accelerating breeding [4,5]. Furthermore, genome-wide DNA marker sets can be used to construct haplotypes for the regions of interest and aid in phylogenetic studies [6,7].

With the emergence and explosive growth of NGS, large amounts of *de novo* assembled genome sequence and resequencing data are being rapidly produced at a low cost. Genome-wide DNA polymorphisms of various strains have been identified by comparison with reference genome sequences [8,9], and genome-sequencing projects using NGS have sequenced not only representative strains of species but also several other strains and identified genome-wide DNA polymorphisms [10–12]. Furthermore, the large genomics projects such as wheat have clarified variation structures among multiple strains using NGS sequencers [13–16]. Integration of these large-scale datasets with datasets generated for individual strains promotes the reuse of data. However, the reliability of these DNA polymorphism datasets varies widely among individual studies because of differences in the quantity and quality of raw data, versions of the reference genomes, data format, and evaluation algorithms. These variations cause difficulty in comparing non-uniform DNA polymorphisms between studies through simple aggregation. Moreover, DNA polymorphism datasets cannot be readily collected because they are frequently distributed from dispersed websites maintained by individual sequencing projects.

DNA polymorphism databases generated for various species enable the study of non-model and model organisms sharing orthologous genes. Currently, certain databases are available that contain the DNA polymorphisms of various species, such as dbSNP [17], Gramene [18], Ensembl Plant [19], and EVA (http://www.ebi.ac.uk/eva/). However, these databases cannot ensure unified experimental and analytical conditions because they merely collect the DNA polymorphism datasets contributed by individual sequencing projects.

Raw NGS data for individual studies can be stored in and retrieved from authorized databanks, such as the DNA Data Bank of Japan (DDBJ) Sequence Read Archive (SRA) [20]. DDBJ SRA data have been exchanged among the DDBJ SRA, the National Center for Biotechnology Information (NCBI) SRA, and the European Bioinformatics Institute (EBI) European Nucleotide Archive (ENA). SRAs contain datasets of several types, such as datasets from whole-genome shotgun (WGS) sequencing, transcriptome analysis, and epigenetics and metagenomics studies. These datasets serve as valuable data sources for further biological big-data mining, and several databases and tools have been developed through the re-analysis of SRA data and made available on distinct websites. For example, the Plant Omics Data Center and ATTED-II contain databases that were developed by re-analyzing gene-expression profiles from transcriptome data in SRAs, and they have generated comprehensive co-expression data from these gene-expression profiles [21,22]. A previous study has presented a conventional pipeline for detecting poly(A) and cluster sites by using the expression information obtained from the re-analysis of transcriptome data in SRAs [23]. Furthermore, epigenetics data in SRAs have been reused: the NCBI Epigenomics database has been constructed as a comprehensive database of whole-genome epigenetic datasets by selecting epigenetics-specific data from the Gene Expression Omnibus and SRAs and re-analyzing these datasets [24], and SraTailor is a software package designed for processing and visualizing epigenetics data in SRAs [25]. However, to the best of our knowledge, no secondary database or tool is currently available for genome-wide DNA polymorphisms in SRAs.

Here, we present DNA polymorphism annotation database (DNApod), an integrated database of genome-wide DNA polymorphisms detected under uniform analytical conditions from NGS-generated WGS datasets in SRAs. This database was developed in order to provide genome-wide DNA polymorphisms of plants, with crop plants being the top priority. In this first study, we describe datasets of rice, maize, and sorghum homozygous single-nucleotide polymorphisms (SNPs) and homozygous insertion or deletion (InDel) polymorphisms that present high potential for serving as DNA markers. The analytical methods are available as a DNApod workflow in the DDBJ Read Annotation Pipeline (DDBJ pipeline) [26] and a virtual machine image. Furthermore, the database facilitates multiple-alignment and phylogenetic-tree analyses performed with the amino acid sequences of orthologous genes by using DNApod genotype datasets and the uploaded original data of users. Moreover, DNApod provides tables of identifier (ID) links between DNApod genotypic data and public phenotypic data. Thus, DNApod holds considerable potential to accelerate studies conducted using genome sequences of multiple species.

## Materials and methods

### Collection of WGS data from SRAs

DNApod genotypic data are re-analyzed WGS datasets extracted from SRAs. To obtain an overview of the registered data on rice, maize, and sorghum in SRAs, we searched the SRAs by using the ENA database search engine [27]. We performed searches by using NCBI taxonomy IDs, including child taxonomy, such as strains. The taxonomy IDs included 4,527, 4,575, and 4,557 IDs of rice, maize, and sorghum, respectively. Next, the sample accessions were counted using a library strategy, such as using the WGS, RNA-seq, and ChIP-seq libraries, which is described in SRA experimental metadata. We applied manual curation to screen WGS libraries out of the SRA samples labeled as OTHER and whole-genome amplification (WGA). Raw NGS reads were downloaded from DDBJ and ENA.

### Construction of uniform-base-quality datasets

SRAs contain datasets of heterogeneous base quality archived as raw NGS data from individual sequencing projects (S1 Fig). From datasets featuring heterogeneous base quality values (QVs), DNA polymorphisms of non-uniform quality might be detected. Therefore, we constructed raw NGS read datasets with unified QVs by using the original perl script of the DDBJ pipeline. First, low-quality bases with QVs in Phred scale under 19 are trimmed from the 5’ and 3’ ends, and trimmed reads with a length under 24 are removed. Finally, trimmed reads for which the ratio of the QV under 14 is over 30% are removed. In the case of a paired-end read, the pair is discarded when one read of the pair is removed in one of the previous steps.

### Detection of DNA polymorphisms

Unified-QV reads were mapped against the reference genome of each species by using Burrows–Wheeler Alignment tool (BWA) ver. 0.6.1-r104 [28] with default options (Table 1), and multiple-mapped reads were removed (i.e., pairs were retained when both reads or one of the reads mapped uniquely, and other pairs were discarded). We detected homozygous SNPs/InDels by using SAMtools mpileup ver. 0.1.18 [29] with default options, bcftools view ver. 0.1.18 with SNP calling (-c), call genotypes at variant sites (-g) and output potential variant sites only (-v), and vcfutils.pl varFilter with a maximal read-depth option of 100 (-D 100). We distinguished homozygous and heterozygous genotypes by using the genotype field (GT) column in variant call format (VCF) [30].

**Table 1:** The versions of the reference genomes and the gene structure annotation.

Organism	Database version
Rice	IRGSP/RAP Build 5 (RAP IRGSP-1.0*) [31]
Maize	Gramene (MaizeSequence.org release-5b) [18]
Sorghum	MIPS/JGI Sbi1.4 [32]

*To enhance user convenience, we mapped DNA polymorphism coordinates from rice IRGSP Build 5 to IRGSP-1.0. Thus, DNApod supports not only IRGSP/RAP Build 5-based but also RAP IRGSP-1.0-based genome-wide DNA polymorphism datasets and known-gene annotations for each DNA polymorphism.

### Known-gene annotation of DNA polymorphisms

SNPs/InDels were annotated and effects for their known gene structure, such as amino acid changes, were predicted by using SnpEff ver. 3.6c (build 2014-05-20) [33]. We created the SnpEff databases by snpEff.jar build command with gene structure information in the general feature format version 3 (GFF3) files. These GFF3 files were generated by extracting coding-sequence features from the GFF3 files distributed by annotation projects (Table 1).

### Visualizing the genomic positions of SNPs and InDels

We visualized the distribution of SNPs and InDels on the reference genome by using our original perl script with the VCF files of homozygous SNPs or homozygous InDels.

### Creating the amino acid sequences

Our original perl script extracts mRNA-coding regions from the GFF3 file and generates mRNA-coding sequences in which reference genome bases at homozygous SNP sites are replaced with bases from a given VCF record, by using “FastaAlternateReferenceMaker” of the Genome Analysis Toolkit (GATK) v3.1–1 [34]. In addition, the perl script converts the nucleotide sequences to amino acid sequences.

### Rice DNA polymorphism coordinate conversion

DNApod provides IRGSP/RAP Build 5-based and RAP IRGSP-1.0-based rice DNA polymorphism datasets. We mapped DNA polymorphism coordinates from rice IRGSP Build 5 to those of IRGSP-1.0. To this end, we first created a FASTA file of the 100 bp flanking each side of the DNA polymorphism in the IRGSP Build 5 genome sequence. This FASTA file was aligned to the genome sequence of IRGSP-1.0 by using BLASTn (BLAST 2.2.31+) [35]. We extracted BLAST results under the following conditions: (1) identity is 100.0%, (2) alignment length equals query length, and (3) query uniquely hits to the target. Finally, we created IRGSP-1.0-based VCFs using our original perl script.

### Validation of homozygous SNPs

SRA datasets have been acquired under different experimental conditions. Thus, they reflect differences in sequence quality and quantity among experiments. From these heterogeneous datasets, DNA polymorphisms are to be detected with uniform reliability. DNApod employs a pre-processing step to filter out low QVs to generate uniform-quality NGS datasets. However, differences in sequence quantity remain an issue. Therefore, we aimed to validate our homozygous SNP detection method with a high-depth and a low-depth dataset. We used the homozygous SNP dataset of the rice line Hitomebore (SRA Sample ID: DRS003820) generated with MutMap [36] as a verified dataset. The Hitomebore NGS dataset was adopted as a representative, high-depth dataset, showing 94.6% coverage and 37.0 depth, in DNApod. Coverage is defined as the percentage of the reference genome bases covered by read alignments. Depth is defined as the average depth of the reference genome bases covered by read alignments. First, to validate the high-depth dataset, we compared the Hitomebore homozygous SNP dataset in DNApod with MutMap data and examined the accuracy rate, which is the concordance rate of genotypes in the common homozygous SNP sites. Next, for low-depth-dataset validation, we constructed a low-depth dataset by random extraction of reads from the Hitomebore dataset and detected the homozygous SNPs. This process was iterated 10 times and the average accuracy rate from the low-depth datasets was determined. Furthermore, we examined the average detection rate, which is the ratio of the number of homozygous SNPs detected from each low-depth dataset to the number of homozygous SNPs in the Hitomebore high-depth dataset.

To check for read loss, we evaluated for each sample the relationship between the percentage of reads removed as multiple-mapping reads and the read length. To this end, we selected samples under the following conditions: (1) paired-end reads, and (2) if a sample accession has some experimental accessions, read length is the same among these experimental accessions. Additionally we calculated the percentage of reads deemed to be multiple-mapping reads.

### Link information between DNApod ID and public phenotypic data

We collected public phenotype data from a 44k SNP set [37], 1536 SNP set [38], Panicle Architecture [39], and High Density Rice Array [40] of the Rice Diversity Project (http://www.ricediversity.org/index.cfm) and National Institute of Agrobiological Sciences (NIAS) [41]. We manually created a table linking the information of DNApod ID (SRA sample ID) with phenotypic data with strain name identification.

### Orthologous analysis

DNApod offers functions for multiple alignment and phylogenetic tree generation with orthologous gene information. We constructed the orthologous gene datasets with protein IDs of annotation databases, which the DNApod genotype database uses (Table 1). The structural definitions of orthologous genes were derived from Plant Genome DataBase Japan [42], which provides ortholog clusters from Reference Sequences (RefSeq) [43] gene annotation. We matched the corresponding RefSeq protein IDs with the corresponding protein IDs of the external database employed by DNApod as follows: (1) We mapped RefSeq protein IDs to external database protein IDs by comparing amino acid sequences by using BLASTP release 2.2.26 [44] with default options. Only BLASTP results with >95% sequence identity for both query (RefSeq) length and target (external database) length were considered. (2) For rice and sorghum, the RefSeq definitions field was described as the external database gene ID. Thus, from the BLAST result, we adopted the protein IDs of the external databases associated with the gene ID described in the RefSeq definition fields. Next, we developed a tool for constructing multiple alignments and neighbor-joining trees on the basis of the orthologous genes using amino acid sequences from the DNApod genotype database, using ClustalW2 ver. 2.1 [45] with 1,000 bootstrap replicates and R package ape version 3.4 [46]. Users can compare amino acid sequences between strains in the DNApod genotype database and their original strain data optionally. When users set the parameter of organism and upload the homozygous SNP data as VCF, which can be prepared using the DNApod workflow, DNApod generates the amino acid sequence file as described under “Creating the amino acid sequences.” DNApod uses implemented as well as user data to generate multiple alignments and neighbor-joining trees.

### System architecture and software

DNApod was implemented on a Linux server by using CentOS release 5.9 (Final) with the following environments: Apache ver. 2.2.31, Tomcat ver. 7.0.67, MySQL ver. 5.7.10, and Java ver. 1.7.0_80-b15.

## Results

### DNApod overview

**Fig 1:**
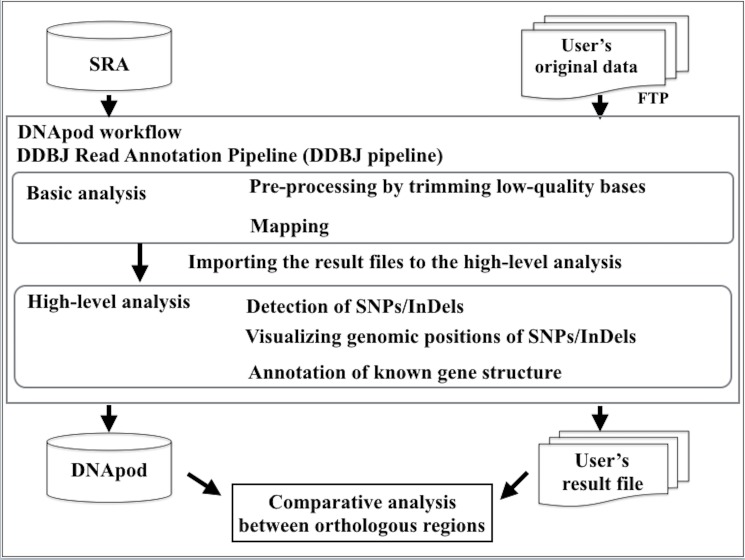
Overview of DNApod. DNApod is used to analyze WGS datasets extracted from NGS data of SRAs. Users can analyze their original data by employing the same process that is used in DNApod and compare orthologous regions between the DNApod genotypic data and their result file.

DNApod genotype datasets comprise DNA polymorphism datasets re-analyzed from NGS data in SRAs by using unified analytical conditions (e.g., uniformity in the quality of raw data, reference genome version, and evaluated algorithms). An overview of the data-generation process for DNApod is presented in Fig 1. The method used for detecting and annotating DNA polymorphisms was implemented as a DNApod workflow, which is a new workflow in the DDBJ pipeline. Users can upload and then analyze their original WGS data by using the graphical user interface of the DDBJ pipeline. The DNApod workflow provides variant call results and supplementary information files, including visualization files showing the distribution of SNPs and InDels in the reference genome, annotation files of known-gene annotations such as synonymous/non-synonymous substitution positions, and amino acid sequence files.

When users seek to change the sensitivity of DNApod genotype datasets, they can reprocess the data to detect homozygous SNPs and homozygous InDels by using the DDBJ pipeline with distinct parameter thresholds. Furthermore, DNApod provides a function for orthologous analysis, which constructs a multiple alignment and phylogenetic tree with amino acid sequences in the DNApod genotype database. Users can upload homozygous SNP data in VCF prepared using the DNApod workflow to compare amino acid sequences between strains in the DNApod genotype database and their original strain data.

### Contents of DNApod genotype datasets

As of April 2016, SRAs contain 10,788, 5,540, and 600 samples for rice, maize, and sorghum, respectively, based on WGS, RNA-seq, and ChIP-seq libraries as well as others (S1 Table). We detected homozygous SNPs and homozygous InDels in WGS data extracted from the SRA. Currently, DNApod holds 1,149 datasets corresponding to 679, 404, and 66 strains of rice, maize, and sorghum, respectively (S1 Table). Because these datasets contain samples of diverse subspecies, germplasms, and experimental sources for each species, in Table 2, we present the DNApod entries according to taxonomic group. DNApod stores information on genomic structural variation, including homozygous SNPs and homozygous InDels. However, this first version of DNApod does not provide information of heterozygous SNPs and heterozygous InDels: It is challenging to detect genome-wide and informative heterozygous SNPs and heterozygous InDels contained in all the DNApod genotypic data because the SRA includes a considerable amount of low-depth data (S3 Fig). The numbers of homozygous SNPs and homozygous InDels in DNApod range from, respectively, 1,074 to 2,558,148 and 136 to 327,684 for rice, 83 to 860,729 and 1 to 648,770 for maize, and 52 to 5,151,219 and 131 to 637,746 for sorghum (Table 2). To validate our detection method, we examined the accuracy rate of homozygous SNP detection in a high-depth and a low-depth dataset. First, we validated our detection method in the high-depth dataset. We compared the rice Hitomebore line in the DNApod genotypic data with MutMap data and examined the accuracy rate for common homozygous SNP sites. DNApod detected 115,895 homozygous SNPs, while MutMap detected 119,042 homozygous SNPs. In total, 100,597 homozygous SNPs were commonly detected by DNApod and MutMap, and 99.997% of the genotypes were concordant. DNApod and MutMap detected 15,298 and 18,445 unique homozygous SNPs, respectively. Next, to validate our method for low-depth data, we constructed 10 low-depth datasets (with an average of coverage 75.8% and depth of 3.1) from the Hitomebore line read dataset in the SRA, detected homozygous SNPs from each low-depth dataset (average number of homozygous SNPs: 50,795), and then compared these to MutMap data. The results showed that an average of 49,013 homozygous SNP sites were in common between a low-depth dataset and MutMap, and the average accuracy rate was 99.998%. The average the detection rate was 43.828%. These results strongly suggest that low-depth data do not influence the accuracy of homozygous SNP detection.

**Table 2:** Current entries of DNApod sorted by subspecies class.

Species ^1^	Subspecies	No. of samples	Coverage, depth	No. of homozygous SNPs per sample	No. of homozygous InDels per sample
*Oryza sativa*	*japonica*	250	19.5, 1.60–96.7, 21.8	1,074–1,342,354	136–174,692
*Oryza sativa*	*indica*	402	21.5, 2.30–92.4, 16.4	76,981–2,412,599	4,680–322,943
*Oryza sativa*		17	23.4, 2.00–88.8, 15.1	38,695–2,321,990	2,058–283,134
*Oryza rufipogon*		5	86.0,16.0–91.7, 15.3	950,660–2,140,218	125,912–268,523
*Oryza nivara*		5	86.3, 15.0–90.5, 14.6	1,638,997–2,558,148	194,857–327,684
*Zea mays*	*mays*	385	0.10, 1.00–91.5, 29.7	83–7,205,121	1–648,770
*Zea mays*	*mexicana*	3	0.50, 1.10–72.5, 7.70	130–7,103,576	6–552,260
*Zea mays*	*parviglumis*	15	26.8, 2.00–72.6, 4.80	458,451–5,352,491	17,441–403,880
*Zea luxurians*		1	26.8, 3.20	860,729	35,526
*Sorghum bicolor*	*bicolor*	53	8.20, 50.6–93.4, 22.9	52–2,278,524	131–324,993
*Sorghum bicolor*	*drummondii*	1	86.2, 19.3	1,708,354	258,027
*Sorghum bicolor*	*verticilliflorum*	2	80.6, 18.0–84.3, 42.0	2,390,239–2,691,724	338,231–387,838
*Sorghum bicolor*		8	86.3, 12.0–91.8, 40.2	257,418–1,701,789	122,536–313,349
*Sorghum propinquum*		2	67.2, 31.5–67.8, 34.9	4,332,194–5,151,219	633,150–637,746

^1^ NCBI taxonomy IDs—*Oryza sativa*: 4530, *Oryza rufipogon*: 4529, *Oryza nivara*: 4536, *Zea mays*: 4577, *Zea luxurians*: 15945, *Sorghum bicolor*: 4558 and *Sorghum propinquum*: 132711

After elimination of multiple-hit reads on the genome, 87% of the DNApod genotypic data showed <5-fold depth with respect to the reference genome (S3 Fig). Low-depth data tend to generate false-negative results. Additionally, we assessed the read loss through removal of multiple-mapping reads. Maize showed higher rates of multi-mapped reads, even at the same read length as that of rice and sorghum (S4 Fig).

The reference genome and annotation versions of rice in DNApod genotype datasets are IRGSP/RAP Build 5. The latest versions for rice, RAP/IRGSP-1.0, have already been released. To enhance user convenience, we have mapped the DNA polymorphism coordinates from rice IRGSP Build 5 to IRGSP-1.0. The number of positions at which DNA polymorphisms were detected on IRGSP Build 5 was 12,982,438, of which 12,802,573 (98.6%) positions were mapped on IRGSP-1.0. Thus, we support rice IRGSP-1.0-based genome-wide DNA polymorphism datasets and known-gene annotations for each DNA polymorphism.

### Overview of the DNApod workflow

**Fig 2:**
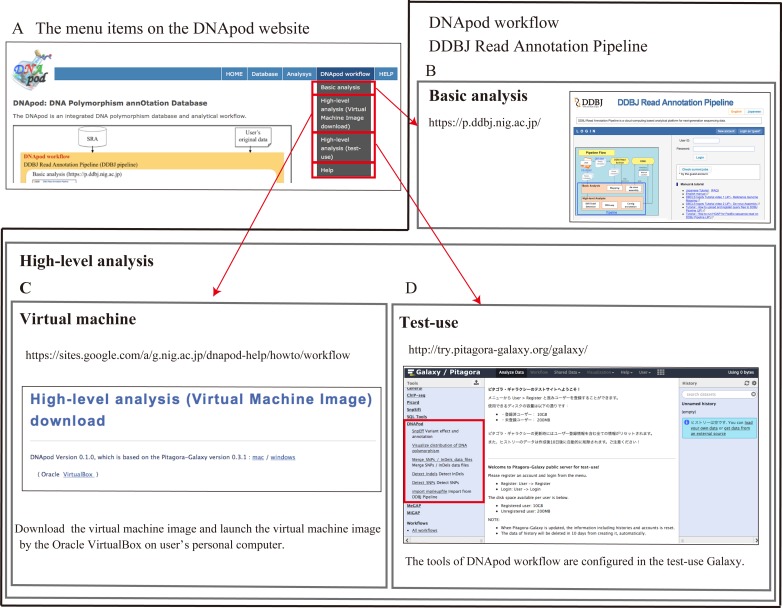
DNApod workflow service configuration. (A) The DNApod workflow can be accessed from the “DNApod workflow” menu on the DNApod websites, (B) The basic analysis is offered as a web service. (C) The high-level analysis is configured in the Galaxy platform, which is implemented in the virtual machine image. Users download this virtual machine image from the DNApod workflow help page and launch the virtual machine image via Oracle Virtualox. (D) Pitagora-Galaxy provides the galaxy server for users to test-use DNApod.

The DNApod workflow is implemented in the DDBJ pipeline, which comprises two analysis components: basic analysis and high-level analysis (Fig 1). In the basic analysis, users can upload their original WGS data to the DDBJ pipeline server by FTP. The user data are pre-processed to remove low-QV sequences and mapped to the reference genomes. The result file from the basic analysis is used as input for the high-level analysis, in which DNApod detects DNA polymorphisms, visualizes their distribution on the reference genome, and annotates them with known gene structures. The DNApod workflow service configuration is shown in Fig 2. The basic analysis is offered as a web service (http://p.ddbj.nig.ac.jp/) (Fig 2B). The high-level analysis is configured in the Galaxy platform, which is implemented in the virtual machine image by Pitagora-Galaxy (http://www.pitagora-galaxy.org/) (Fig 2C). The respective tools for high-level analysis are encapsulated in the Docker container (https://www.docker.com), and Galaxy runs these Docker containers (S2 Fig). Users download the virtual machine image from DNApod workflow help page and launch it through Oracle VirtualBox (https://www.virtualbox.org) on their personal computer (S2 Fig). Furthermore, users can test-use the high-level analysis on the Pitagora-Galaxy server (http://try.pitagora-galaxy.org/galaxy/) (Fig 2D).

### DNApod components and web interface

#### Main components

DNApod includes four components: “Genotype database,” “DNApod workflow graphical user interface,” “Phenotype database,” and “Orthologous analysis.” The use of these components is described in the “Help” menu in DNApod.

#### Genotype database

**Fig 3:**
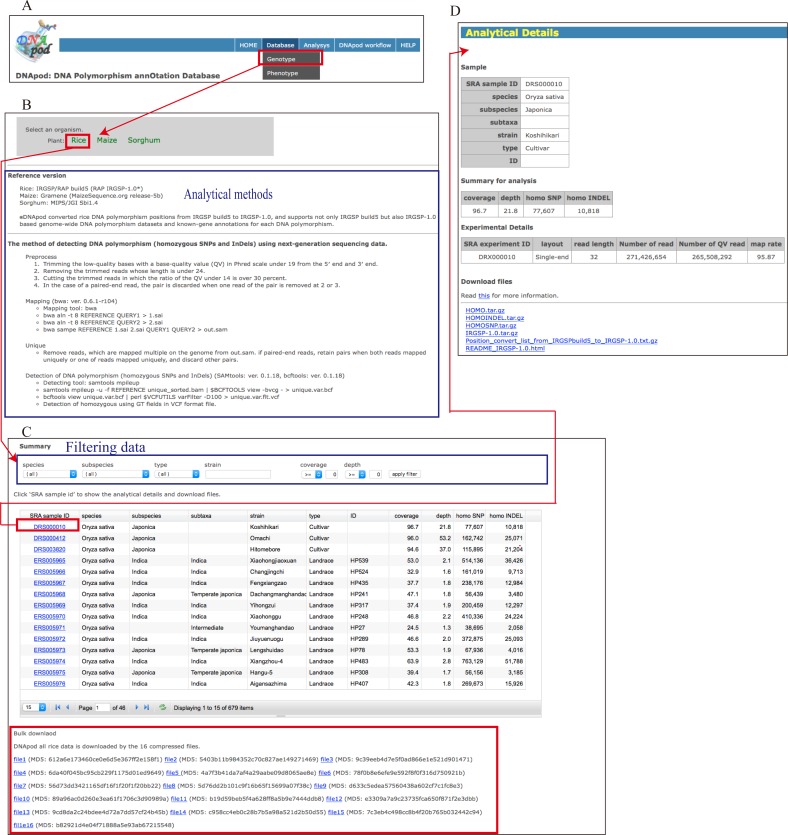
Statistics and analytical information of DNApod genotypic data. (A) Menu items leading to the genotype database and DNApod workflow, (B) “Select an organism” screen, (C) “Summary” screen, and (D) “Analytical Details” screen.

The genotype database is accessible from menu items (Fig 3A). The “Select an organism” screen is displayed (Fig 3B), and the analytical method used for detecting homozygous SNPs and InDels is indicated in this screen. The “Summary” screen is displayed upon selection of an organism, which can be surveyed in this screen; the summary includes the species, subspecies, and strain names, coverage, depth, and numbers of homozygous SNPs and InDels (Fig 3C). Data can be filtered by species and subspecies names and type, such as cultivar, wild accession, or landrace, strain name, coverage, and depth. Users can bulk download the data per organism. For additional information and data download, an “Analytical Details” screen is displayed when “SRA sample ID” is clicked; this screen presents the information described in the “Summary” screen and information regarding experiments: SRA experiment ID, layout (such as paired- or single-end layout), read length, number of reads, number of QV-filtered reads, and map rate (Fig 3D). Users can download data for DNA polymorphisms, including variant call files, visualization files showing the distribution of SNPs/InDels on the reference genome, known-gene annotation files for each DNA polymorphism such as synonymous/non-synonymous substitution positions, and the amino acid sequence files. Variant call files are supplied in VCF, a versatile format used by various genome browsers, such as Integrative Genomics Viewer [47].

#### DNApod workflow graphical user interface

Users can analyze their own NGS data under the curative conditions of the DNApod workflow through a DNApod graphical user interface. The DNApod website describes the DNApod method for detecting DNA polymorphisms, including parameter settings (Fig 2B). The workflow can be accessed from the menus on the DNApod website (Fig 2A). The DDBJ pipeline (DNApod workflow) basic analysis is accessible from “Basic analysis” in the menu (Fig 2B). The virtual machine image for high-level analysis can be downloaded from “High-level analysis (Virtual Machine Image download)” in the menu (Fig 2C). Test runs of high-level analysis can be executed from “High-level analysis (test-use)” in the menu (Fig 2D). Furthermore, the DNApod workflow has a detailed help page (Fig 2A), which provides the DNApod workflow overview, a high-level analysis (virtual machine image) download link, the DNApod workflow (DDBJ pipeline basic analysis and high-level analysis) manual, and trial data.

#### Phenotype database

**Fig 4:**
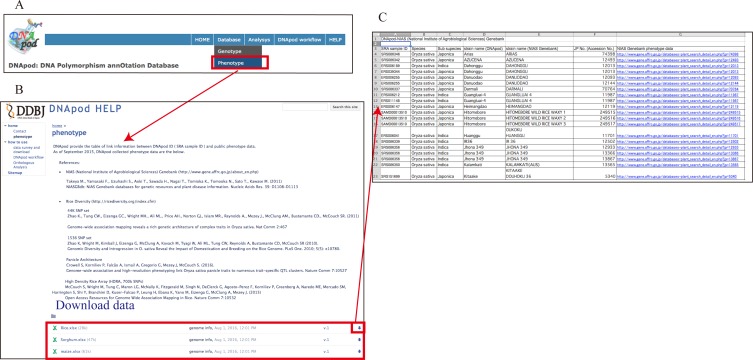
Phenotype link information. (A) Menu item leading to the phenotype link database, (B) “Phenotype” database screenshot, (C) a downloaded table of linked information between DNApod IDs (SRA sample IDs) and public phenotypic data.

The phenotype database is accessible from the menu items (Fig 4A). DNApod has been collecting public phenotypic data, and distributing the table of linked information between DNApod IDs (SRA sample IDs) and phenotypic data. As of April 2016, DNApod genotypic data linked to phenotypic information included 29 rice samples linked to 44k SNP set, 29 rice samples to 1536 SNP set, 13 rice samples to Panicle Architecture, and 22 rice samples to High Density Rice Array of the Rice Diversity Project (http://www.ricediversity.org/index.cfm). Moreover, DNApod contains the phenotypic information for 28 rice samples, 26 maize samples, and 6 sorghum samples linked to National Institute of Agrobiological Sciences (NIAS) Genebank (Fig 4B). Link information can be downloaded (Fig 4C).

#### Orthologous analysis

**Fig 5:**
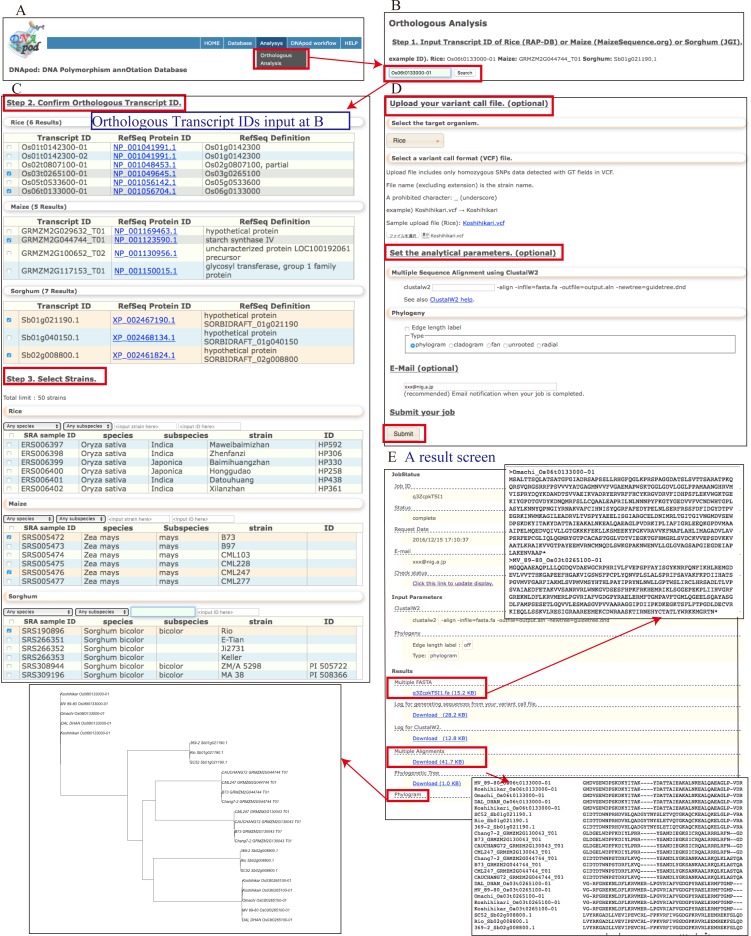
Function of “Orthologous Analysis” in DNApod. (A) Menu item for an “Orthologous Analysis,” (B) setting parameters: transcript ID, (C) setting parameters: transcript IDs in orthologous groups of the transcript specified in (B) and strains, (D) setting parameters: original user data and analytical parameters, and (E) a result screen.

Orthologous region alignment is accessible from the menu (Fig 5A). When a transcript ID of rice, maize, or sorghum is input, the information for the orthologous group of the specified transcript is displayed together with a RefSeq protein ID and RefSeq definition (Fig 5B and 5C). Transcripts and strains are selected as query data for the orthologous analysis. Strains can be filtered by species, subspecies, and strain names and the ID, which is the accession ID of the resource center. If a user uploads own homozygous SNP data in VCF prepared by the DNApod workflow, DNApod facilitates amino acid sequence comparison based on DNApod genotypic data and the user data (Fig 5D). Moreover, if necessary, users can set the analytical parameters such as ClustalW2 and Phylogeny. Thus, users can obtain multiple FASTA files as a query file, result information files consisting of multiple-alignment files, and tree-image files (Fig 5E).

## Discussion

We have developed DNApod, a readily reusable database of genome-wide DNA polymorphisms featuring homogeneous reliability; the database was developed by detecting DNA polymorphisms under unified analytical conditions by using WGS datasets extracted from SRAs. DNApod currently describes homozygous SNPs/InDels and known-gene annotations for these polymorphisms in rice, maize, and sorghum; the polymorphisms can be used as DNA markers. DNApod provides an analytical workflow for analyzing user NGS data and for orthologous analysis. DNApod is a collection of manually curated public phenotypic data, which are linked to DNApod IDs (SRA sample IDs).

SRA datasets have been acquired under varying experimental conditions that have included differences in sequence quality and quantity among experiments. To detect DNA polymorphisms with uniform reliability from SRA WGS datasets featuring non-uniform quality, DNApod performs a pre-processing to filter out low QVs and then detects DNA polymorphisms by using a uniform method with the same threshold. However, the matter of sequence quantity remains unresolved. The DNApod genotypic data present diverse depths of coverage after the removal of multiple-hit reads on the reference genome, starting from a minimum of one-read depth; 87% of the DNApod genotypic data present a <5-fold depth on a reference genome (S3 Fig). Low-depth data might generate false-negatives during the detection of DNA polymorphisms. We investigated the relationship between the number of reads lost by removing multiple-hit reads and read length. Even when the read lengths were the same, maize showed a markedly lower rate of lost reads in total reads after pre-processing for QV than did rice and sorghum (S4 Fig). The subfamilies Panicoideae (sorghum and maize) and Ehrhartoideae (rice) branched from a common ancestor 50 million years ago (MYA), and sorghum and maize diverged 13.5 MYA [48]. Paleopolyploidy in Panicoideae and Ehrhartoideae occurred following a genome polyploidization event 70 MYA. Subsequently, maize underwent a tetraploidization event, immediately after which numerous chromosomal breakages and fusions resulted in a return to the diploid state 12–15 MYA [49,50]. Sorghum has not undergone a genome polyploidization event since 70 MYA [32]. Therefore, maize shows a large syntenic block covering 89% of the genome [51], and this large-scale syntenic block would cause higher multiple-hit reads than in the case of rice and sorghum. Polyploidy is widespread among plant species. In soybean, multiple rounds of duplication and diploidization occurred in the genome [52]. In banana, almost all cultivars are triploid [53], and bread wheat is hexaploid [54]. In this study, we examined the effect of genome polyploidization events on the lost read rate by removing the multiple-mapping reads only for maize. However, genome polyploidization events might increase the lost read rate and genome regions of that cannot be analyzed by the removal of multiple-hit reads in the genome. If a sample that has undergone genome polyploidization events is sequenced, the experimental design should focus on read length than on read quantity. Furthermore, the method for removing multiple-hit reads is better to be improved.

In studies conducted using low-depth data, genotype imputation is employed [55,56]. Genotype imputation with haplotype patterns helps with the prediction of uncertain genotypes, and certain tools have already been developed and used for genotype imputation [57–59]. DNApod should validate the most relevant methods for genotype imputation. This imputation strategy will facilitate the detection of heterozygous SNPs and InDels and correction of DNA polymorphisms misdetected because of low-QV reads. When low-depth genotype datasets are employed with genotype imputation, DNApod can provide high-density DNA markers on the genome. This may contribute to the discovery of responsible genes by GWAS and more accurate phylogeny estimation. In the future, new versions of reference genomes and known-gene annotations from respective reference databases will be released at an accelerated pace for both model and non-model organisms. Thus, we plan to update the version of reference genomes and known-gene annotations in order to enhance the reliability of the DNApod genotypic data. Furthermore, in DNApod, we plan to provide a function for developing DNA markers, such as Cleaved Amplified Polymorphic Sequence, by using the DNApod genotypic data.

We have been collecting phenotypic information. In this study, DNApod collected phenotypic information from the NIAS Genebank and Rice Diversity Project, including environmental data such as phenotyping regions and years. This information contributes to the analysis of environmental and phenotypic data. For almost all of DNApod genotypic data, the phenotypic information provided was incomplete. Phenotypic and genotypic information is necessary for breeding programs and GWAS; thus, we anticipate that DDBJ, NCBI, and EBI will systematically collect both phenotypic and genotypic information in the future.

Public SRA data are increasing drastically [60]. As of March 2016, the number of WGS entries, which was specified in the SRA study type, was 29,125; this is only the number of studies, and thus the number of samples will be higher. With this increase of SRA data, DNApod requires to be steadily updated, and the scope of DNApod will be expanded to cover organisms from bacteria to plants. Thus, DNApod will potentially serve as a valuable data-science infrastructure element for breeding studies and GWAS by using a combination of phenotypic and geographic data. Moreover, DNApod will promote the efficient secondary use of public, open-access data.

## Supporting information

S1 Fig Heterogeneous base-quality raw sequence reads in SRAs. SRAs contain data of various quality values among NGS datasets from individual projects. To detect DNA polymorphisms with uniform reliability, DNApod performs pre-processing to filter out low quality values and detects DNA polymorphisms by using a uniform threshold.
(TIF)

S2 Fig Overview of the Galaxy virtual machine. The high-level analysis is configured in the Galaxy platform, which is implemented in the virtual machine image. The virtual machine image of the high-level analysis is launched by the Oracle VirtualBox on the user’s personal computer. The respective tools in high-level analysis are encapsulated in the Docker container, and Galaxy runs these Docker containers to execute the job.
(TIFF)

S3 Fig Data quantity of each sample. Data quantity is described as the depth after the removal of multiple-hit reads on the genome. The depth of a reference genome is <5-fold in 87% of the DNApod genotypic data.
(TIF)

S4 Fig Read loss per read length caused by elimination of multiple-hit reads. Maize exhibits a more profound effect resulting from read loss than do rice and sorghum after the elimination of multiple-hit reads. This predicted that a large-scale syntenic block of maize would cause comparatively higher multiple-hit reads.
(TIF)

S1 Table Sample number of registered SRA and DNApod by study type. Data as of April 2016. The sample number of the registered SRA was searched using ENA. “Library strategy” is explained on the DDBJ SRA website (http://trace.ddbj.nig.ac.jp/dra/submission_e.html).
(DOCX)
