# Supplementary figures and images for: DNApod: DNA polymorphism annotation database from next-generation sequence read archives
Source: PLoS One. 2017 Feb 24;12(2):e0172269. doi: 10.1371/journal.pone.0172269 (PMC5325239; doi:10.1371/journal.pone.0172269)

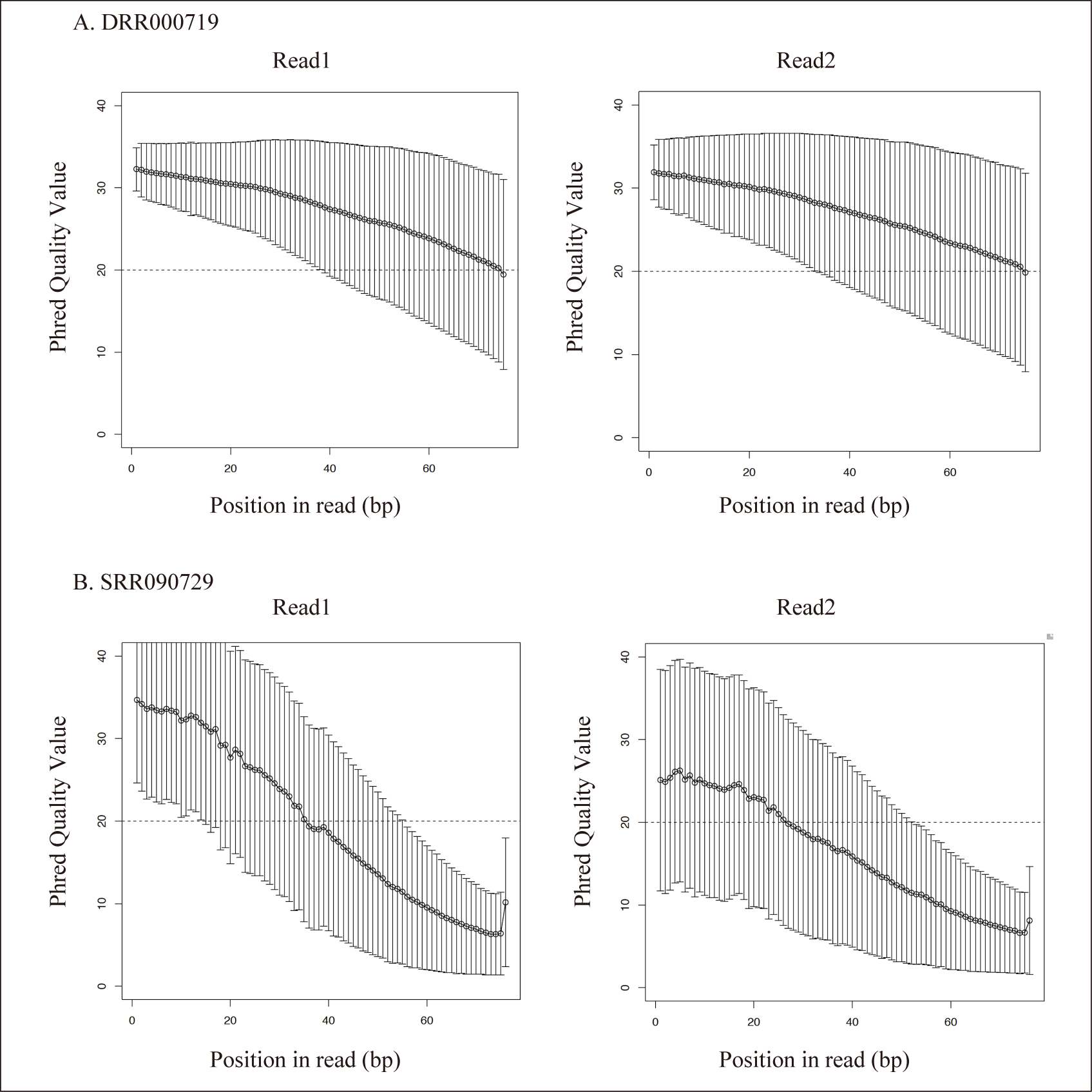

Supplement: S1 Fig — SRAs contain data of various quality values among NGS datasets from individual projects. To detect DNA polymorphisms with uniform reliability, DNApod performs pre-processing to filter out low quality values and detects DNA polymorphisms by using a uniform threshold. (TIF) [file pone.0172269.s001.tif]

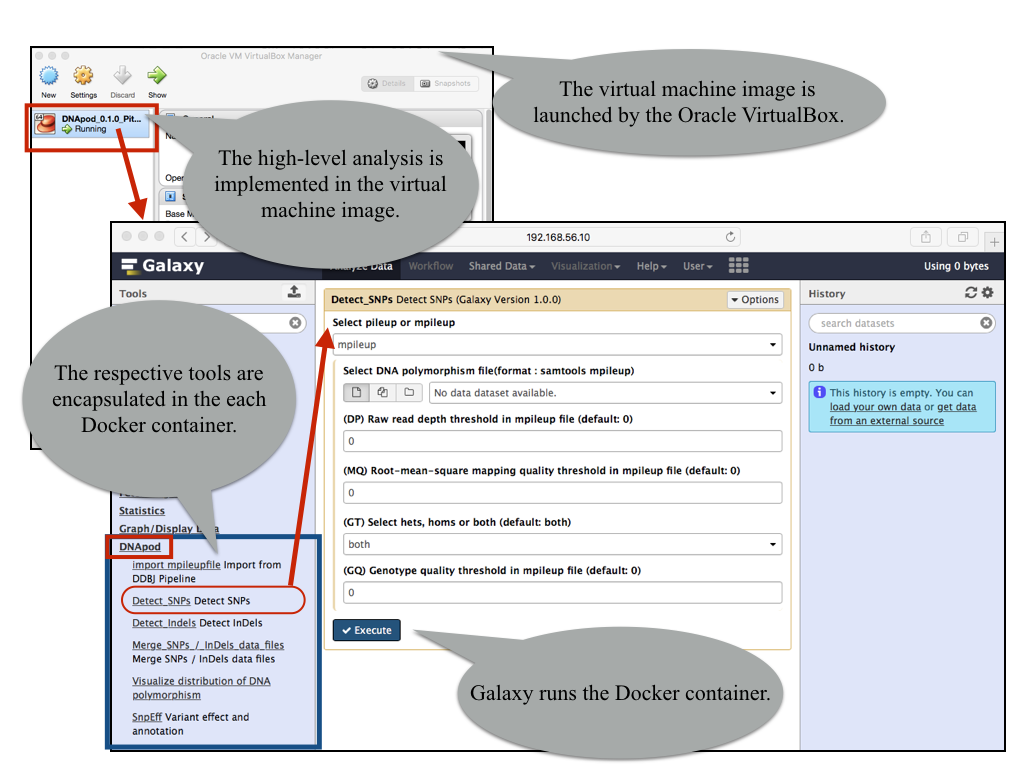

Supplement: S2 Fig — The high-level analysis is configured in the Galaxy platform, which is implemented in the virtual machine image. The virtual machine image of the high-level analysis is launched by the Oracle VirtualBox on the user’s personal computer. The respective tools in high-level analysis are encapsulated in the Docker container, and Galaxy runs these Docker containers to execute the job. (TIFF) [file pone.0172269.s002.tiff]

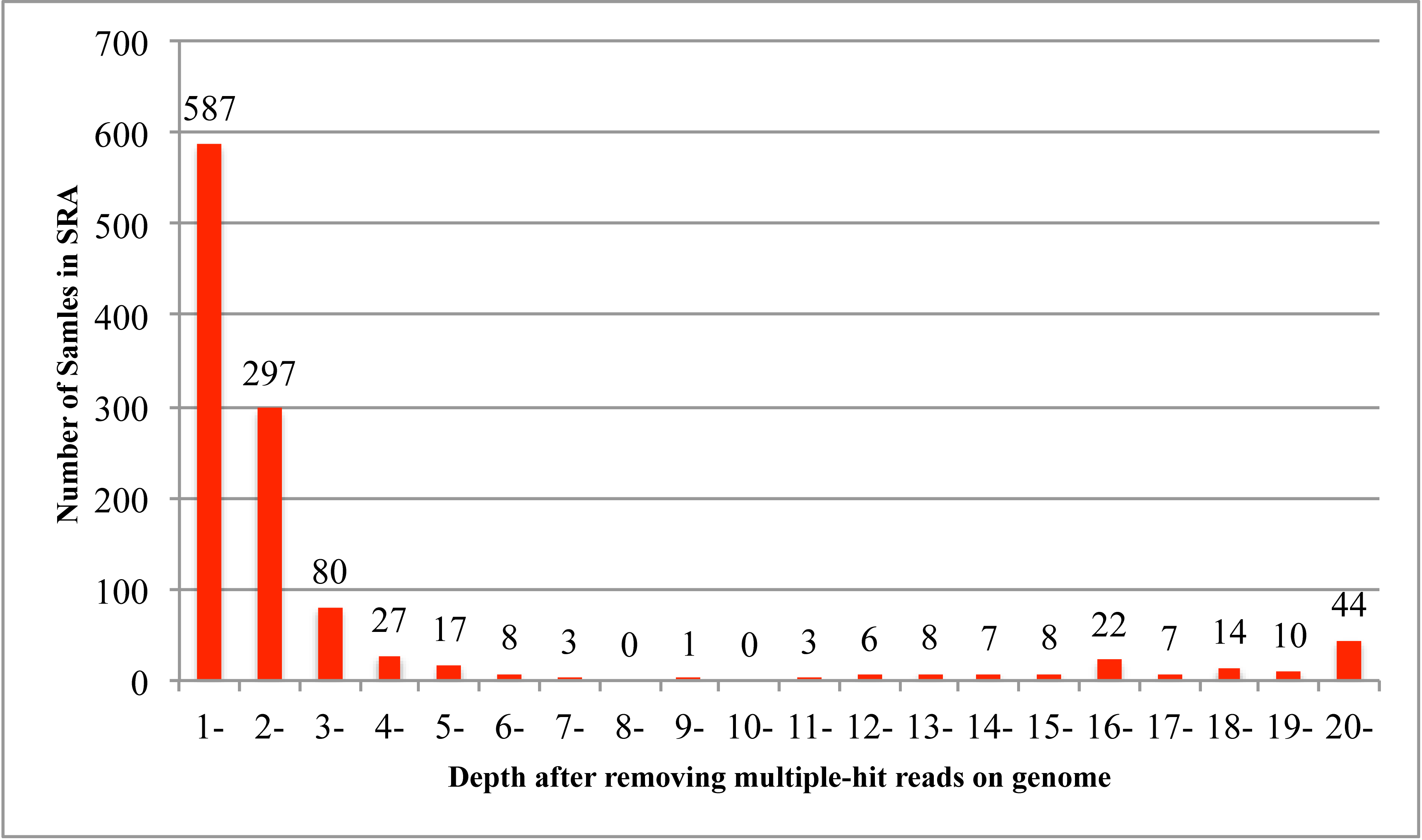

Supplement: S3 Fig — Data quantity is described as the depth after the removal of multiple-hit reads on the genome. The depth of a reference genome is <5-fold in 87% of the DNApod genotypic data. (TIF) [file pone.0172269.s003.tif]

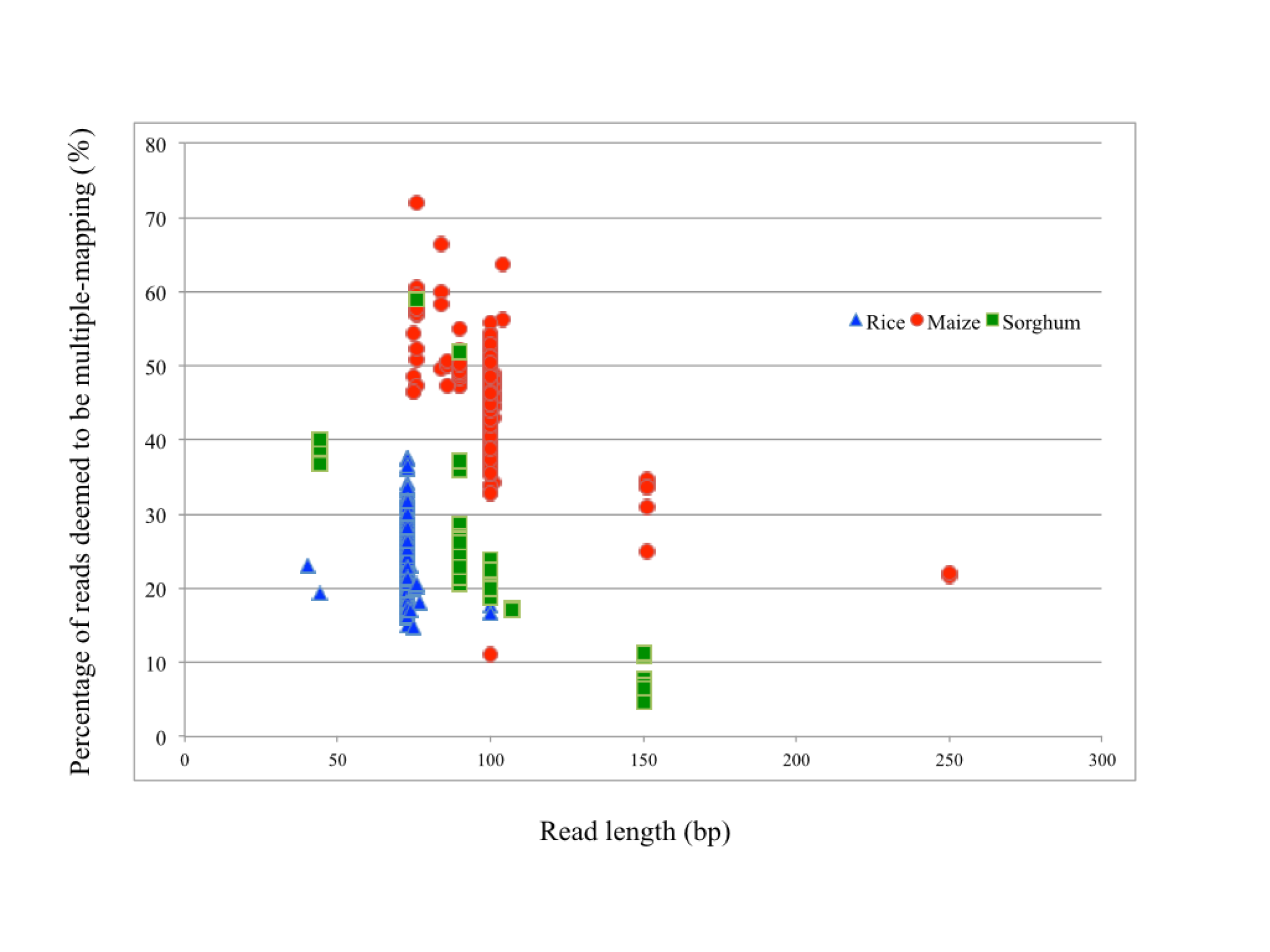

Supplement: S4 Fig — Maize exhibits a more profound effect resulting from read loss than do rice and sorghum after the elimination of multiple-hit reads. This predicted that a large-scale syntenic block of maize would cause comparatively higher multiple-hit reads. (TIF) [file pone.0172269.s004.tif]
