# Supplementary material for: DNApod: DNA polymorphism annotation database from next-generation sequence read archives
Source: PLoS One. 2017 Feb 24;12(2):e0172269. doi: 10.1371/journal.pone.0172269 (PMC5325239; doi:10.1371/journal.pone.0172269)
Supplement: S1 Table — Data as of April 2016. The sample number of the registered SRA was searched using ENA. “Library strategy” is explained on the DDBJ SRA website (http://trace.ddbj.nig.ac.jp/dra/submission_e.html). (DOCX) [file pone.0172269.s005.docx]

**S1 Table. Sample number of registered SRA and DNApod by study type**

(A) Rice

| **Library strategy** | **Sample number of registered SRA** | **Sample number of registered DNApod** |
| --- | --- | --- |
| AMPLICON | 23 | 0 |
| Bisulfite-Seq | 126 | 0 |
| ChIP-Seq | 67 | 0 |
| CLONE | 1 | 0 |
| DNase-Hypersensitivity | 5 | 0 |
| EST | 3 | 0 |
| FL-cDNA | 2 | 0 |
| MeDIP-Seq | 14 | 0 |
| miRNA-Seq | 90 | 0 |
| MNase-Seq | 7 | 0 |
| ncRNA-Seq | 13 | 0 |
| OTHER | 808 | 100 |
| RNA-Seq | 1,924 | 0 |
| WGA | 1 | 0 |
| WGS | 7,620 | 579 |
| WXS | 84 | 0 |
| Total | 10,788 | 679 |

(B) Maize

| **Library strategy** | **Sample number of registered SRA** | **Sample number of registered DNApod** |
| --- | --- | --- |
| AMPLICON | 17 | 0 |
| Bisulfite-Seq | 79 | 0 |
| ChIP-Seq | 129 | 0 |
| CLONE | 1 | 0 |
| EST | 3 | 0 |
| FINISHING | 1 | 0 |
| FL-cDNA | 16 | 0 |
| MeDIP-Seq | 2 | 0 |
| miRNA-Seq | 29 | 0 |
| MNase-Seq | 5 | 0 |
| MRE-Seq | 8 | 0 |
| ncRNA-Seq | 82 | 0 |
| OTHER | 213 | 28 |
| RNA-seq | 4,357 | 0 |
| WGA | 101 | 0 |
| WGS | 497 | 376 |
| Total | 5,540 | 404 |

(C) Sorghum

| **Library strategy** | **Sample number of registered SRA** | **Sample number of registered DNApod** |
| --- | --- | --- |
| AMPLICON | 1 | 0 |
| Bisulfite-Seq | 2 | 0 |
| EST | 2 | 0 |
| FL-cDNA | 3 | 0 |
| miRNA-seq | 5 | 0 |
| OTHER | 6 | 0 |
| RNA-seq | 224 | 0 |
| WGA | 1 | 1 |
| WGS | 356 | 65 |
| Total | 600 | 66 |

Data as of April, 2016. The sample number of registered SRA was searched using ENA. An explanation of the ‘Library strategy’ can be found on the DDBJ SRA website (http://trace.ddbj.nig.ac.jp/dra/submission_e.html).
